# Analysis of the Effect of the Magnetic Field on Water Flux Through TIP3;1 Aquaporins Using Molecular Dynamics in GROMACS

**DOI:** 10.1007/s00232-025-00368-x

**Published:** 2026-02-05

**Authors:** Diego Fernando Nieto-Giraldo, José Mauricio Rodas Rodríguez, Javier Torres-Osorio

**Affiliations:** 1 Department of Chemistry, Universidad de Caldas, Calle 65 # 26-10, Manizales-Caldas, Colombia; 2 Department of Physics, Universidad de Caldas, Calle 65 # 26-10, Manizales-Caldas, Colombia

**Keywords:** Magnetobiology, Molecular dynamics, Computational chemistry, Osmotic permeability, Aquaporins

## Abstract

Magnetobiology studies the effects of magnetic fields on biological systems. In this context, the Magnetobiology Research Group at the University of Caldas has hypothesized that magnetic treatment may influence water flow through cell membranes. To contribute to evaluating this hypothesis, this study analyzes the effect of magnetic fields on water transport through TIP3;1 aquaporins of *Zea mays* L. Molecular dynamics simulations were performed using a modified version of GROMACS that includes magnetic forces in the Velocity–Verlet algorithm, exposing a TIP3;1 homotetramer embedded in a lipid bilayer to static, uniform magnetic flux densities (B) ranging from 0 to 10 T and oriented along the membrane normal (z-axis). The system was solvated with the SPC/E water model. To assess the impact on water flow, we analyzed the conformational dynamics of TIP3;1, protein–water interactions within the single-file channel, and the osmotic permeability coefficient (p_f_). Trajectory analysis revealed that the magnetic field alters the average pore radius and increases protein conformational variability. These structural changes affect the intermolecular interactions between the protein and water molecules, influencing water mobility through the channel. Systems exposed to magnetic fields showed up to a threefold increase in pf compared to the control. These findings suggest that magnetic fields can modulate water flow through membranes, supporting a possible mechanism of magnetically influenced water transport in biological systems.

**Graphical Abstract:**

Water transport through TIP3;1 aquaporins from *Zea mays* was simulated using a modified version of GROMACS under magnetic fields (B = 0–10 T). Results show that B modifies the pore structure and protein–water interactions, increasing osmotic permeability (p_f_) up to threefold. These findings suggest a possible magnetic modulation of membrane water flow and a mechanism for magnetoreception.
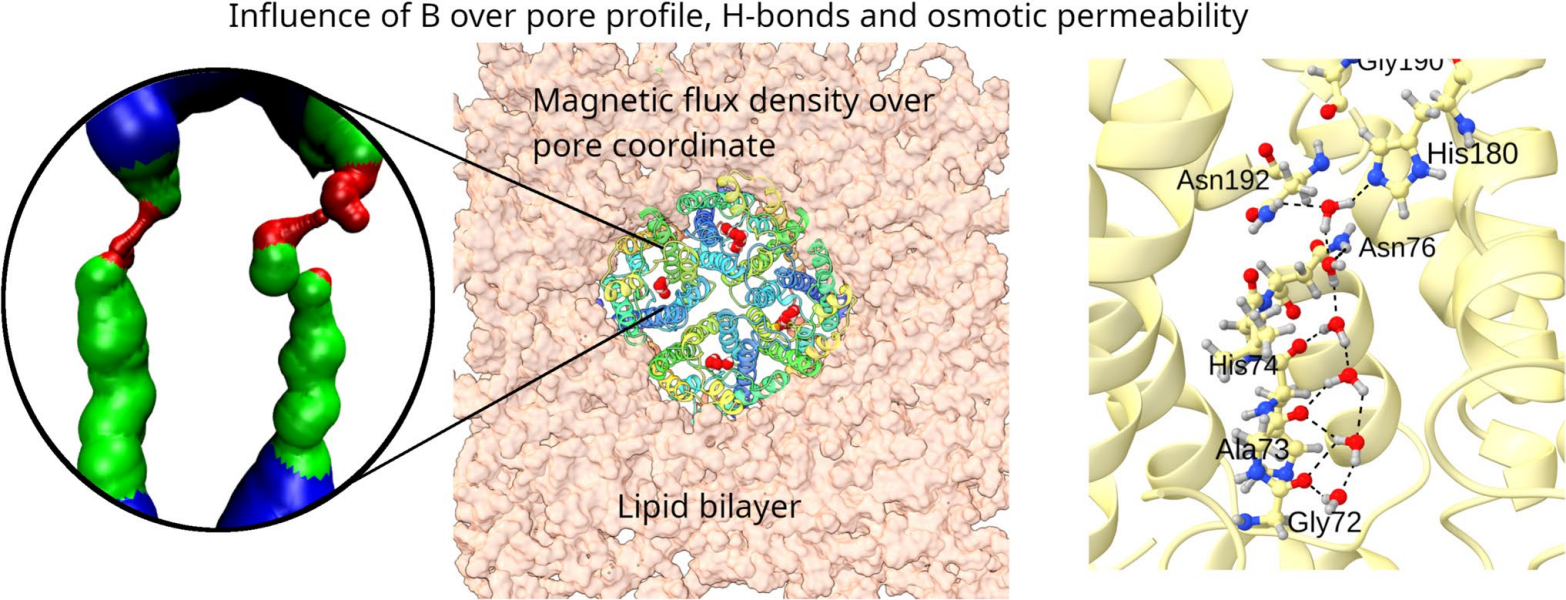

**Supplementary Information:**

The online version contains supplementary material available at 10.1007/s00232-025-00368-x.

## Introduction

Magnetobiology is defined as the scientific study of magnetic field effects on biological systems, encompassing both permanent magnets and electromagnets. Recent advancements in this field have catalyzed the development of innovative agronomic techniques and promising applications across biotechnology and medicine (Maffei 2014; Belyavskaya 2004; Betti et al. 2011; Cakmak et al. 2010; Mahajan and Pandey 2014; Paul et al. 2006). In the context of plant organisms, magnetic treatment has been observed to exert a profound influence on a variety of physiological and cellular processes. These include a reduction in germination time, enhanced protoplast fusion, augmented chlorophyll content, stimulated cell and root growth, and increased seed dry mass accumulation (Fischer et al. 2004; Yamashita et al. 2004; Radhakrishnan and Kumari 2013; Nedukha et al. 2007; Xu et al. 2013, 2012; Sakhnini 2007).

The influence of magnetic fields has been demonstrated to extend beyond plant systems, encompassing various physiological processes in animals, including modulation of neural activity, regulation of hormone secretion, and modification of immune response (Betti et al. 2011; Mahajan and Pandey 2014; Panagopoulos et al. 2024; Xie 2022; Binhi and Rubin 2022). It has been documented that a number of species possess magnetoreception capabilities, which enable orientation and navigation based on geomagnetic cues. Examples of species exhibiting such capabilities include migratory birds, sea turtles, fish, and certain insects. These phenomena are frequently ascribed to magnetite-based receptors or radical pair dynamics within photoreceptor proteins, thereby underscoring the cross-kingdom relevance of magnetic field effects and emphasizing the necessity for understanding their molecular and cellular mechanisms (Binhi 2021; Dempsey 2021; Hart 2024).

Despite the relatively modest energy contribution of magnetic fields in comparison to thermal fluctuations, a preponderance of both experimental and theoretical evidence suggests their capacity to modulate biological systems through specific mechanisms. These include the radical pair mechanism, magnetite-based sensing, and alterations in ion transport or membrane permeability (Gerhards et al. 2025; Binhi 2025; Krylov and Osipova 2023). The radical pair hypothesis posits that biochemical reaction sensitivity to weak magnetic fields is quantum-chemically founded, thereby modifying singlet–triplet interconversion rates in photoreceptor proteins, such as cryptochromes (Karki et al. 2021; Thoradit et al. 2023). While these mechanisms have been associated with physiological responses in both plant and animal systems, the precise molecular pathways remain largely unresolved. Membrane proteins, such as aquaporins, represent potential targets for modulation.

In biological or experimental contexts, synchronized or focused magnetic fields of higher intensity can arise in laboratory devices such as coils, solenoids, or Helmholtz setups used for seed treatment, and by ferromagnetic particles that locally enhance the field. These experimental systems provide reproducible platforms to study magnetic effects on biomolecules. Although the present study uses static, uniform magnetic fields as an idealized model, it is conceptually related to these controlled experimental exposures.

The Magnetobiology Research Group has documented significant reductions in seed germination time following exposure to exogenous magnetic fields. An investigation of this phenomenon revealed that magnetically treated seeds exhibited elevated levels of gibberellic acid (GA_3_), a phytohormone that plays a critical role in the disruption of dormancy and the initiation of germination (Rivera Giraldo 2016). These findings lend support to the hypothesis that magnetic fields enhance water flow into vacuoles, thereby promoting the hydrolysis of inactive gibberellin forms stored within these organelles and facilitating accelerated germination processes.

A comprehensive understanding of water movement between vacuoles and cytoplasm in magnetically treated seeds necessitates a thorough examination of aquaporin function, as these proteins play a pivotal role in regulating water and solute transport across cellular membranes. TIP3;1 aquaporins, a subset of tonoplast intrinsic proteins, have been demonstrated to be particularly relevant to seed development through their exclusive expression in seed vacuoles and their cessation of expression upon germination initiation (Footitt et al. 2019; Gattolin et al. 2011; Lee et al. 2020). The present study proposes and evaluates the hypothesis that magnetic fields influence water flow through TIP3;1 aquaporins, potentially contributing to the observed increases in GA_3_ concentration and subsequent reductions in germination time (Novikova et al. 2014).

Finally, it should be noted that water is a diamagnetic substance; however, previous molecular and experimental studies have reported measurable effects of magnetic fields on its self-diffusion coefficient and hydrogen-bond network. These effects may occur indirectly through modifications of the dielectric environment or through interactions with charged or polar groups in proteins. Consequently, aquaporins, which form the principal water channels across membranes, constitute excellent model systems for exploring how external magnetic fields could influence transmembrane water transport.

In order to evaluate this hypothesis, the present investigation utilizes molecular dynamics simulations to characterize the effects of magnetic fields on water flow through *Zea mays* L. TIP3;1 aquaporins. A modified GROMACS software version was employed, incorporating magnetic field effects on particle motion and interactions. Implementation and validation procedures for this version have been described in detail in our previous publication in Nieto-Giraldo et al. (2025a). The analysis examines how magnetic fields with flux densities ranging from 0 to 10 T influence TIP3;1 conformational dynamics, protein-water interactions within the pore structure, and osmotic permeability coefficients.

Molecular dynamics simulations are inherently constrained to nanometer-scale systems and nanosecond timescales, while biological magnetic field responses typically occur across macroscopic spatial dimensions over extended exposure periods. To address this disparity and ensure effective magnetic stimulus application under simulation conditions, an additional high-intensity magnetic field of 100 kT was evaluated, the selection process and rationale for this high-field condition are detailed in the Methods section.

## Methodology

### Protein Modeling

Since the experimental structure of TIP3;1 from *Zea mays* has not been elucidated, its three-dimensional conformation was predicted using AlphaFold2, based on the amino acid sequence retrieved from UniProt (ID: Q9ATL7). AlphaFold2 has been extensively validated for aquaporins and related membrane proteins, showing structural deviations below 2 Å when compared to homologous experimental structures, which supports its reliability for the TIP3;1, as demonstrated in our previous study (Nieto-Giraldo et al. 2025b). The predicted monomeric structure was then assembled into a homotetramer using the GalaxyWeb server, following the canonical arrangement observed in other plant aquaporins (Seok et al. 2021; Shin et al. 2014; Ko et al. 2012; Wu et al. 2014).

### System Construction

The tetrameric complex was inserted into a POPC/POPE/CHL1 lipid bilayer using the CHARMM-GUI Membrane Builder, ensuring correct alignment along the membrane normal with the PPM server (Jo et al. 2009; Feng et al. 2023; Berendsen et al. 1995). The embedding process employed the replacement method, in which the lipid region is first represented by spheres whose positions guide the random placement of 210 POPC, 205 POPE and 100 CHL1 molecules around the protein. The resulting membrane patch spanned approximately 15 × 15 nm in the XY-plane. A cubic simulation box was defined with periodic boundary conditions in all directions, ensuring at least 1.2 nm between the protein surface and the box edge to prevent artefacts. The system was then solvated in a 15 × 15 × 9 nm water box using the SPC/E model (note that TIP3;1 refers to the aquaporin protein, not the water model), and counterions (Cl⁻) were added to achieve charge neutrality. The final assembled system contained approximately 215,000 atoms.

### System Equilibration

All molecular dynamics simulations were performed with our modified version of GROMACS 2023.2 using the CHARMM36 force field for both the protein and lipid components. The simulation protocol comprised two phases (Abraham et al. 2015; Spoel et al. 2005; Gowers et al. 2016). First, an equilibration stage of 5 ns was carried out under an NPT ensemble, employing a simulated annealing protocol with a gradual temperature increase from 0 to 303.15 K. Temperature and pressure were regulated using the V-rescale thermostat and Parrinello–Rahman barostat, respectively. To prevent bilayer distortion during density adjustments, a harmonic positional restraint of 1 kcal mol⁻^1^ Å⁻^2^ was applied along the membrane normal during equilibration. Following equilibration, a production phase of 5 ns was performed without restraints. Detailed simulation setup in the supplementary information (SI).

### Magnetic Field Implementation

A modified version of GROMACS was used to incorporate the effects of static magnetic fields in molecular dynamics simulations by introducing an additional magnetic term into the Velocity–Verlet integrator. The modification allows the computation of the Lorentz-type force, influencing particles with non-zero charges and velocities.

#### Validation of the Magnetic-Field Implementation

The modification to the Velocity–Verlet algorithm used to include magnetic forces was validated previously (Nieto-Giraldo et al. 2025a). In brief, the validation comprised three test problems:

i Diffusion of monovalent ions in a periodic water box under static magnetic fields, recovering the expected changes in ionic mobility;
ii Calculation of the self-diffusion coefficient of bulk water (SPC/E) in cubic boxes exposed to fields between 0 and 10 T, showing consistent trends with previous molecular dynamics reports; and.
iii Structural stability tests (RMSD and radius of gyration) of a solvated lysozyme molecule under fields between mT and kT, confirming numerical stability of the integrator and physical consistency of trajectories.

The full implementation details and numerical tests are provided in Nieto-Giraldo et al. (2025a).

#### Magnetic Field Geometry

In all simulations, the external magnetic field was static, spatially uniform, and aligned with the membrane normal (z-axis). The same field orientation and uniformity were used for every replicate and intensity reported in this study. Finally, seven conditions were simulated: 0 T, 1 T, 2 T, 4 T, 6 T, 10 T, and 100 kT (extreme case). Each condition was executed in duplicate, yielding a total simulated time of 140 ns (10 ns × 2 replicates × 7 treatments). The use of the extreme 100 kT field was motivated by magnetic dose equivalency arguments, as discussed in section "Magnetic Dose Considerations in Molecular Dynamics Studies".

### Trajectory Analysis

The analysis of molecular dynamics trajectories was designed to evaluate the functional behavior of TIP3;1 under different magnetic field intensities, focusing specifically on structural parameters and water permeation coefficient.

#### Pore Radius Profile

First, the conformational profile of the channel was characterized by computing the pore radius along the central axis of the conduction pathway, performed using the HOLE2 module integrated with the MDAnalysis library. A reference coordinate was defined based on the oxygen atom of a water molecule positioned within the channel lumen at the end of the equilibration phase. From this origin, a set of equidistant points was generated along the pore axis (z-axis), and for each point, HOLE2 determined the maximum radius of a virtual sphere that avoids steric clashes with protein atoms, using their van der Waals radii. This procedure enabled the construction of a continuous mean pore profile for each simulation condition, and to extract average and standard deviation values that quantify the conformational fluctuations of the pore (Michaud-Agrawal et al. 2011; Smart et al. 1993, 1996; Rapaport 1983).

#### Hydrogen Bonding

In parallel, the hydrogen bonding network between the protein and permeating water molecules was examined using both the Hydrogen Bond Analysis and Hydrogen Bond Autocorrelation tools from MDAnalysis. These analyses allowed us to compute the average hydrogen bond frequency, providing insight into the dynamic interaction landscape within the single-file region of the channel (Zhu et al. 2004).

#### Water Diffusion and Osmotic Permeability

Finally, water mobility across the membrane fragment was assessed through calculation of the p_f_. For this purpose, the water diffusion coefficient (D_n_) was determined with Visual Molecular Dynamics (VMD), and the p_f_ was calculated using the numerical relationship between the two coefficients described by Zhu et al. (2004) and Hashido et al. (2007), p_f_ = v_w_ * D_n_. In this equation, v_w_ correspond to the volume of one water molecule (Humphrey et al. 1996; Giorgino 2019; Yeh and Mou 1999). The determination of the D_n_ followed the Einstein relation for one-dimensional diffusion along the channel axis (z-axis), D(τ) = MSD(τ)/2Eτ, where E = 1 for a single coordinate analysis and τ is the lag time.

The analysis was restricted to water molecules occupying the single-file region of the channel for each monomer, which was defined based on structural limits obtained from the HOLE2 pore profile. Only water molecules residing in this region during the simulation interval were considered. For each selected water molecule, the MSD as a function of lag time was computed by averaging the squared displacement of its position along the z-axis. Subsequently, a linear fit was applied to the MSD versus τ data in the diffusive regime, and the slope was used to calculate D_n_ with VMD. This approach ensures that D_n_ reflects the effective diffusion along the pore axis, excluding transient confinement effects. Finally, the computed D_n_ was combined with the molar volume of water to obtain the p_f_, which serves as an indicator of water transport efficiency under the different magnetic field conditions tested.

Full simulation parameters and analysis scripts are provided in the Supplementary Information (SI).

### Limitations

It should be emphasized that the CHARMM36 force field and SPC/E water model do not include explicit magnetic terms in their Hamiltonian. Magnetic effects were introduced through the modified integrator rather than by altering potential energy functions. Consequently, polarization or induced magnetization effects are not represented, and results must be interpreted as qualitative and exploratory. Despite these limitations, the observed structural and dynamic trends are consistent with physical expectations and provide insight into possible mechanisms by which magnetic fields could modulate water transport in biological membranes.

## Results and Discussion

The results are presented in two sections, the analysis of the system exposed to magnetic fields with B between 0 and 10 T, and the effects observed under a field with B of 100 kT, which will be presented in the following section.

### Description of the Study System

**Fig. 1:**
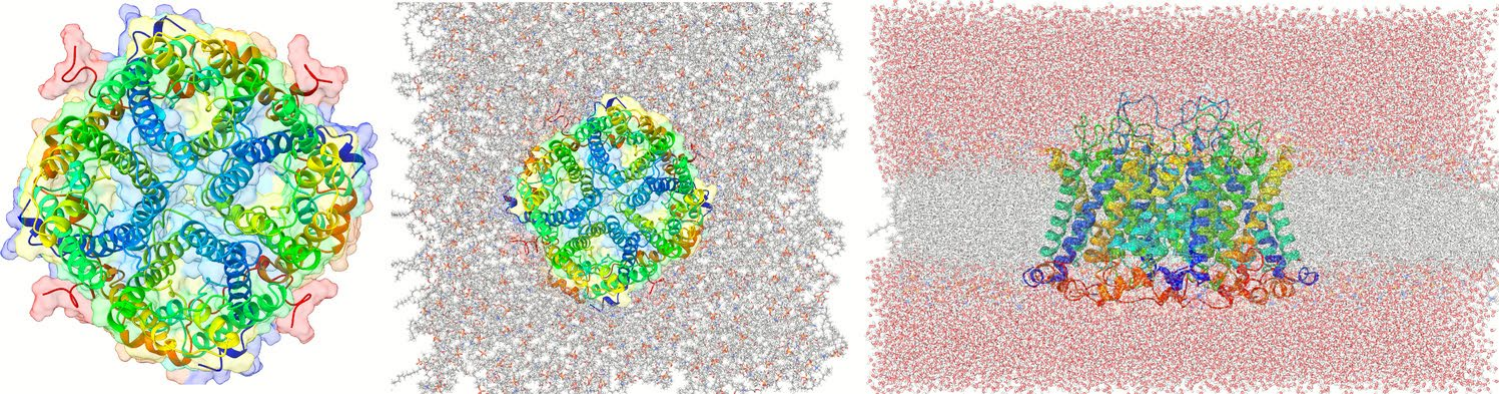
Description of the construction of the study system. **a** Homotetrameric complex consisting of TIP3;1 monomers obtained from Alphafold2. **b** Homotetramer immersed in a lipid bilayer fragment composed of POPC. **c** Initial system for simulations

**Fig. 2:**
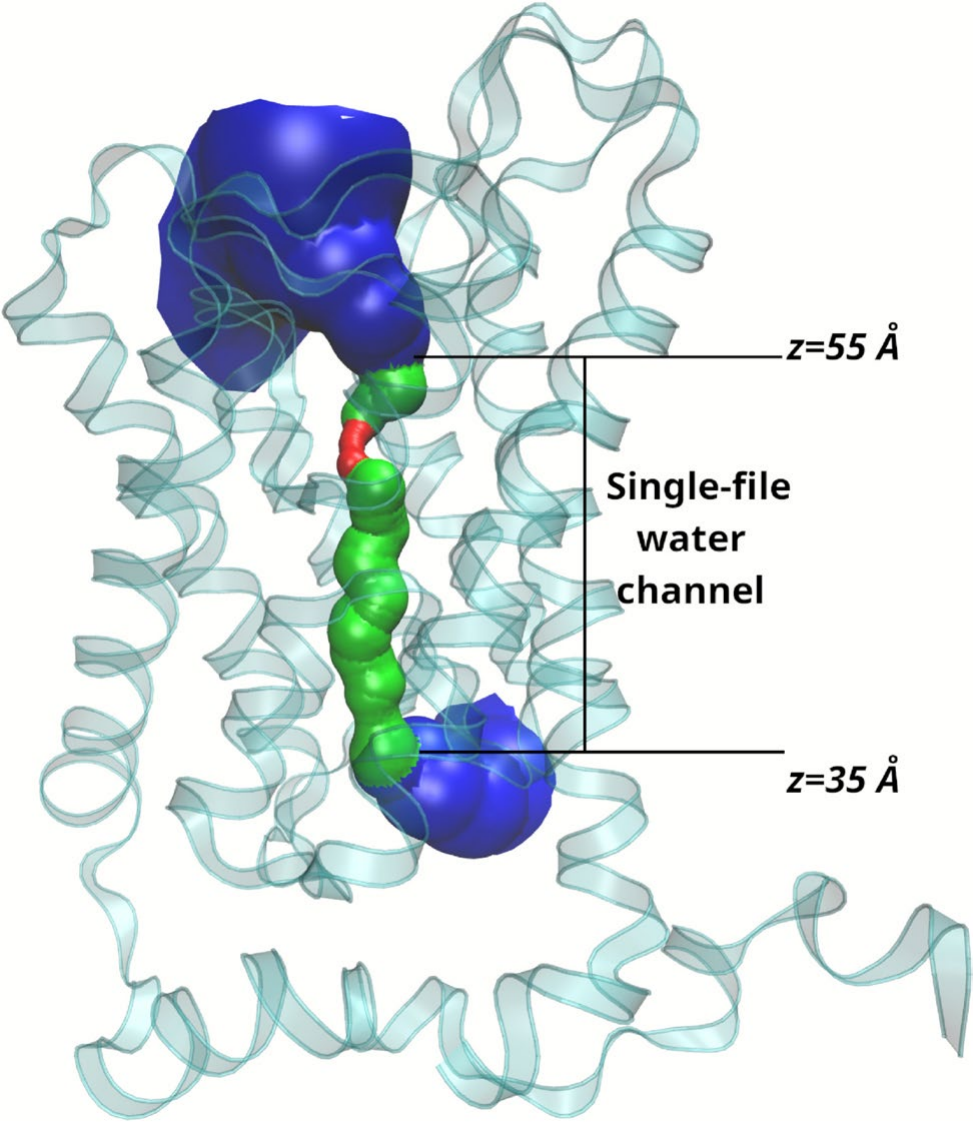
Representation of the single-file water channel in the study system

The system consists of 214,688 atoms, including the TIP3;1 homotetramer, the lipid bilayer and 46,255 water molecules (Fig. 1). The analysis focuses on the flow of water through the single file water channel, a region of each monomer that allows the passage of a single row of water molecules at a time. As water moves through this region, it interacts with amino acid residues in the channel and exhibits different behaviour to the rest of the water molecules. In the system, the single file water channel is located between 35 and 55 Å of the pore coordinate (z) (see Fig. 2).

### Water Flux Through TIP3;1 at B Between 0 and 10 T

By exposing the system under study to magnetic fields of magnitude B between 0 and 10 T, the effects on each parameter were observed and are detailed below.

#### Conformational Dynamics of TIP3;1

**Fig. 3:**
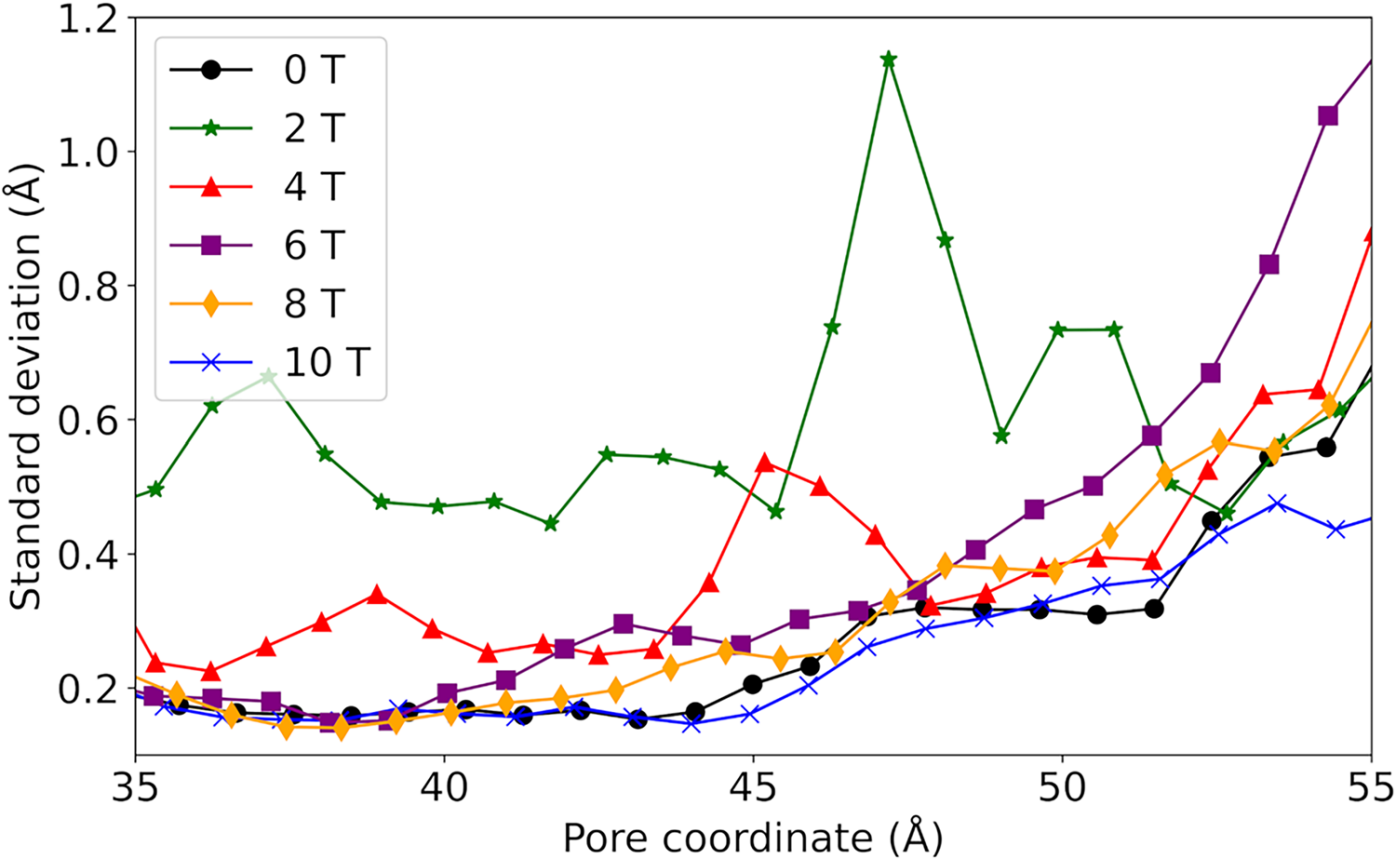
Standard deviation of pore radius for systems exposed to B between 0 and 10 T

The structural behavior of the TIP3;1 tetramer remained stable throughout all simulation conditions, with no significant deviations in secondary structure elements. Furthermore, the standard deviation of the pore radius profiles (Fig. 3) did not reveal structural disruption or unfolding even at the highest magnetic field intensities, confirming that the system remained within a physically realistic regime during the simulations.

Although no large-scale denaturation was detected, magnetic field exposure increased the amplitude of conformational fluctuations in loop regions and pore-lining residues. These fluctuations modulated the transient pore geometry, thereby affecting the radius and continuity of the single-file water pathway. A gradual increase in pore radius variability was observed between 2 and 6 T, suggesting that moderate magnetic intensities enhance conformational flexibility without compromising structural integrity.

**Fig. 4:**
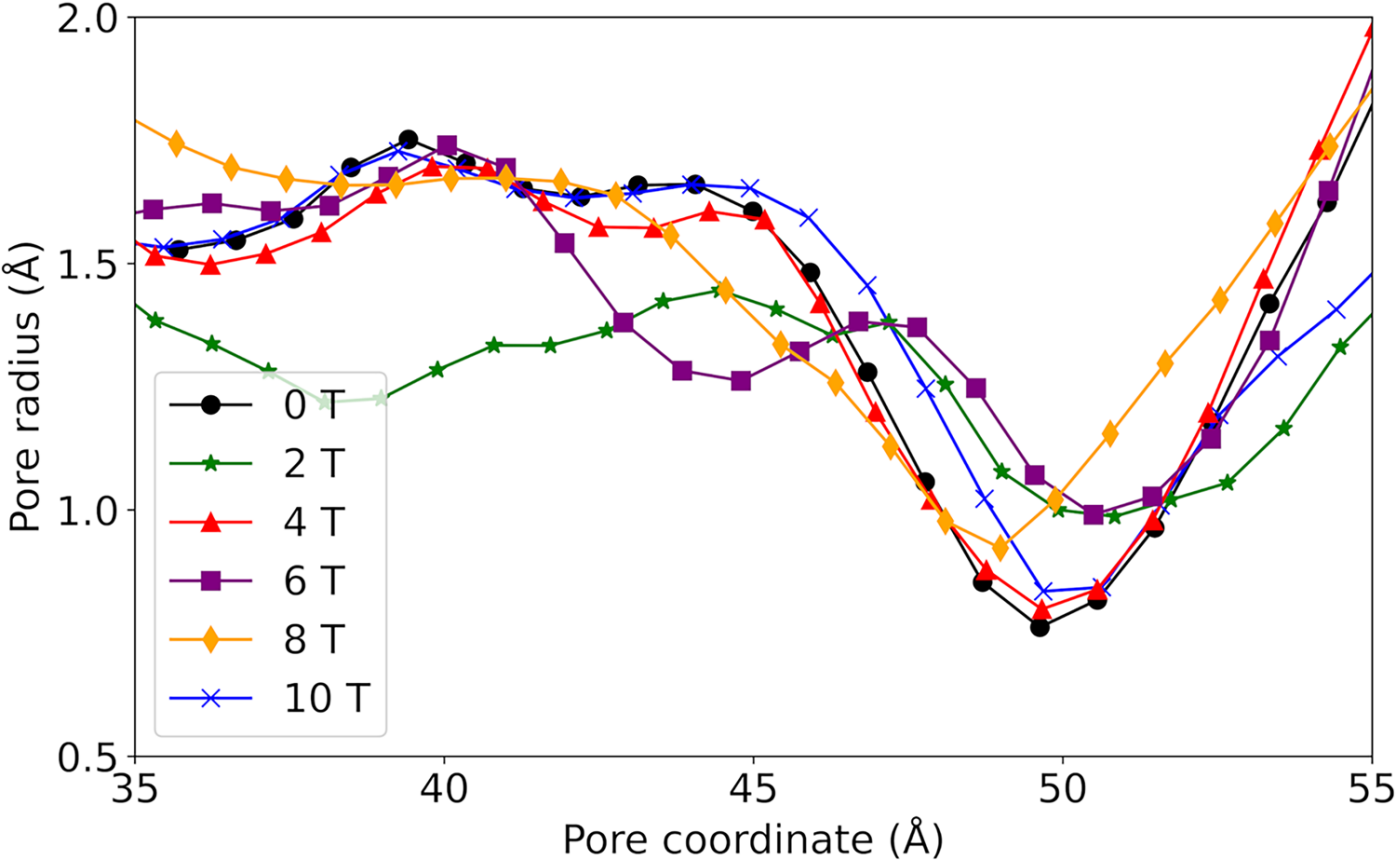
Average pore radius resulting after exposing the study system to magnetic fields with B between 0 and 10 T

Analysis of the average pore radius (r) shows consistent patterns across the systems studied (Fig. 4). A r of about 1.5 Å is observed between the 35 and 45 Å pore coordinate, with the bottleneck of the channel close to the extracellular region at about z = 50 Å. However, the r at the channel bottleneck is up to twice as large in the systems exposed at 2 T and 6 T compared to the other systems. In addition, between 35 and 45 Å of the pore coordinate, the system exposed to B of 2 T has a smaller pore radius than the other systems (1.25 Å), indicating a greater magnitude influence of the field at B of 2 T.

The pore radius profiles revealed two distinct responses depending on field intensity (Fig. 4). Low and moderate fields (2–6 T) induced wider and more dynamic pores, while higher intensities (> 10 T) slightly reduced the mean radius, likely due to transient conformational compaction. These changes were accompanied by variations in the hydrogen-bond network between water molecules and the pore-lining residues.

The average hydrogen-bond frequency increased under all magnetic conditions compared to the control, suggesting enhanced polarization of the water molecules and stronger transient interactions with the protein surface.

The standard deviation of the pore radius (Fig. 3) shows minimum values between 35 and 45 Å at z, gradually increasing towards the extracellular region in most treatments. This trend suggests that the maximum conformational changes occur near the bottleneck to facilitate water passage (Nieto-Giraldo et al. 2025b). In general, the systems exposed to the magnetic field have a larger standard deviation than the control, and in particular the system exposed to B of 2 T has more than twice the standard deviation of the control along the entire pore coordinate.

Thus, changes in protein conformational behaviour indicate that the magnetic field influences pore size and dynamics over the simulation time, with effects of greater intensity for systems exposed to B between 2 and 6 T, and with a smaller influence for B from 8 to 10 T. In addition, variations in pore size in the simulation can affect the patterns of protein-water intermolecular interactions in the single file water channel and the water mobility in this region.

#### Intermolecular Interactions

**Fig. 5:**
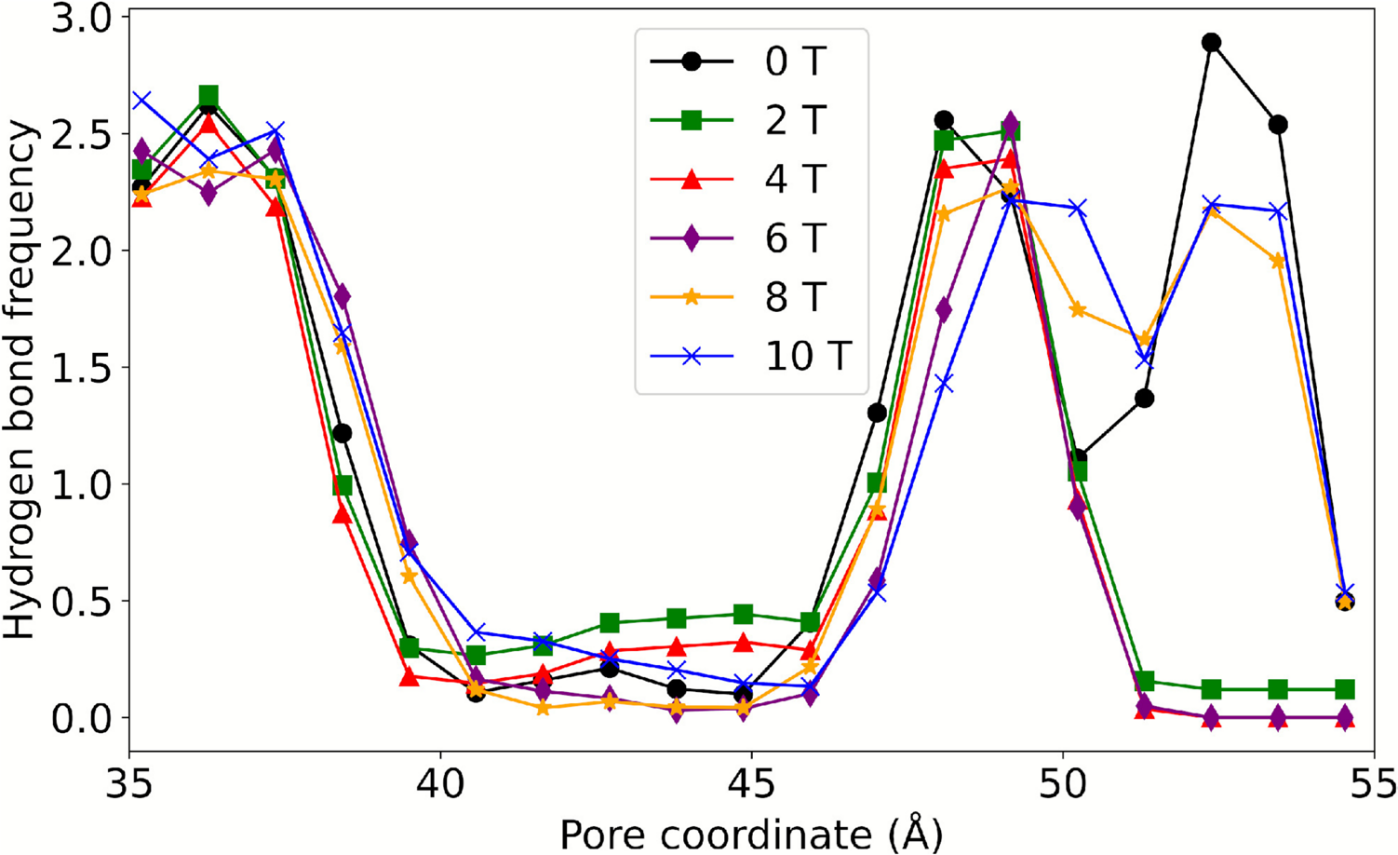
Frequency of protein–water hydrogen bonds along the pore coordinate for systems exposed to B between 0 and 10 T

**Fig. 6:**
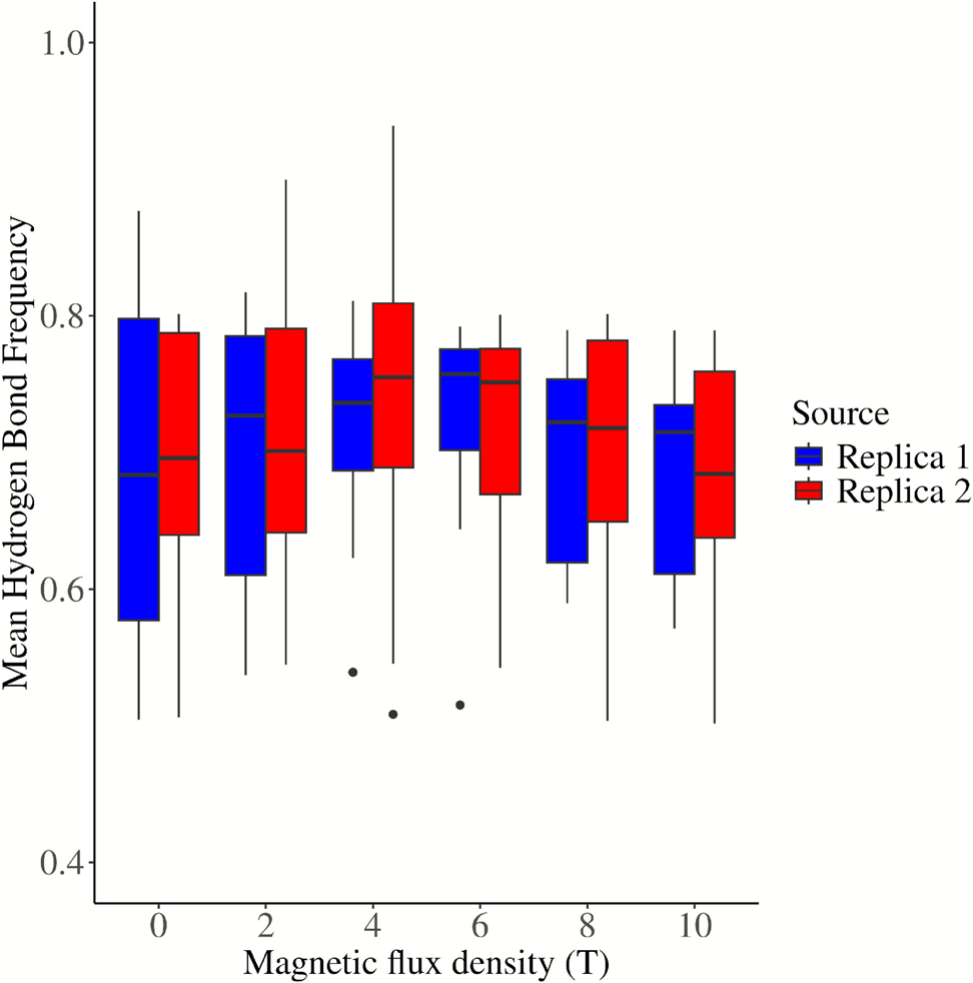
Frequency of protein–water hydrogen bonds as a function of B

The protein-water hydrogen bond frequency distribution (see Fig. 5) demonstrates a peak near the intra- and extracellular regions in all simulations. Systems exposed to 2 T, 4 T, or 6 T exhibit a minimum frequency between 51–54 Å, while the remaining systems demonstrate a peak in this region. Furthermore, Fig. 6 reveals that the hydrogen bonding frequency distribution in both replicates increases in systems exposed to B from 2 to 6 T, and then gradually decreases until reaching a value similar to the control system at 10 T. These variations in hydrogen bonding frequency could affect the mobility of water, since an increase in these interactions could reduce the freedom of molecular motion as suggested by Khajeh et al. (2020) and Yan et al. (2024).

#### Water Flux

**Fig. 7:**
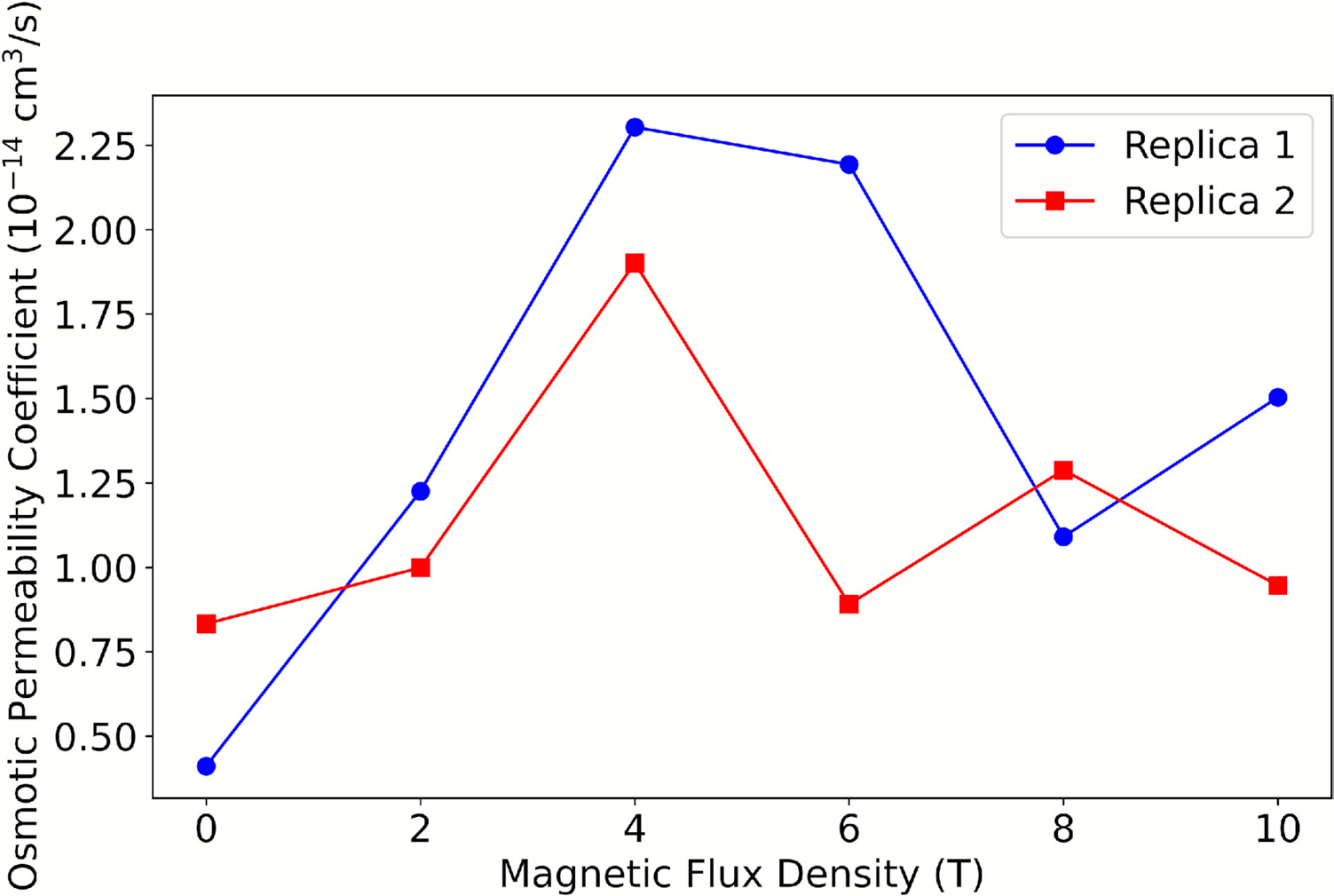
p_f_ coefficient as a function of B

Conversely, the mobility of water through the membrane fragment is also influenced by the magnetic field. This phenomenon is evident because the permeation coefficient p_f_ increases threefold compared to the control system upon exposure to a magnetic field of 2 T to 6 T. However, the effect diminishes in systems exposed to B of 8 T and 10 T (see Fig. 7).

**Fig. 8:**
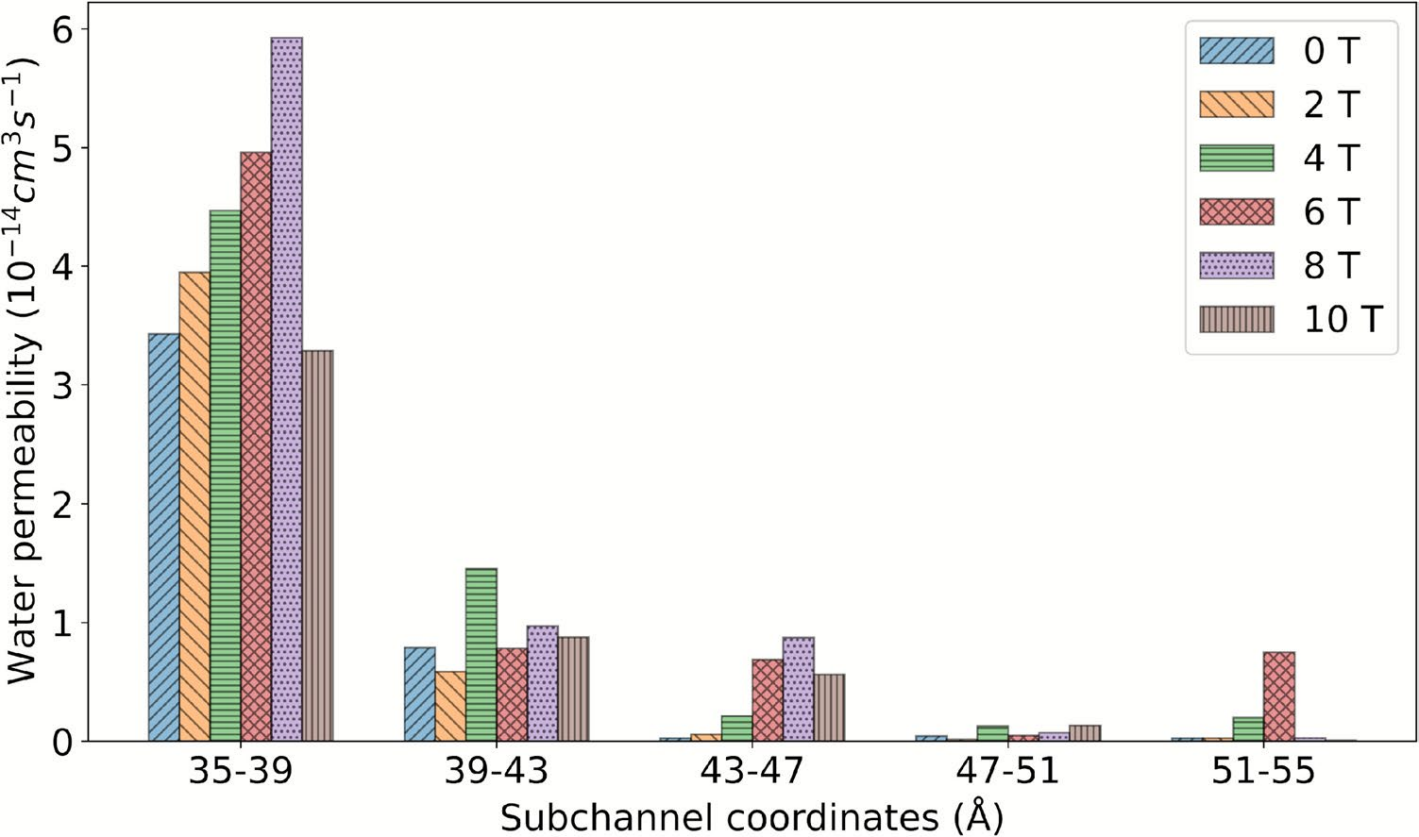
Osmotic permeability coefficient as a function of B for the five subchannels of the AQP

Moreover, Fig. 8 illustrates that the magnetic field exerts a more pronounced effect on water mobility for the three subchannels located in proximity to the intracellular region. Conversely, the effect diminishes for the two subchannels that are proximate to the extracellular region. This is attributable to the presence of the channel bottleneck, which impedes water mobility and engenders a uniform and low p_f_ across all systems.

Although a higher number of hydrogen bonds is often associated with reduced water mobility, our results indicate that magnetic fields simultaneously increase both hydrogen bonding and osmotic permeability (p_f_). This apparent paradox can be explained by the interplay between hydrogen bonding and conformational dynamics. Magnetic perturbations may alter the dynamics of charged and polar side chains, affecting the local dielectric environment and facilitating larger-amplitude fluctuations in pore-lining residues. These transient widening events dominate the water transport process, compensating for the local mobility reduction that stronger hydrogen bonding would typically impose. Thus, an increased hydrogen-bond frequency can coexist with enhanced overall water flux, as transiently wider pores allow faster single-file rearrangements of hydrogen-bonded water molecules.

This interpretation is consistent with previous studies showing that field-induced conformational flexibility can modulate aquaporin conductance, as demonstrated for the electric-field–dependent behavior of the *Aqy1* aquaporin (Rahimi and Lohrasebi 2020).

In summary, the effect on protein conformational dynamics, intermolecular interactions, and water mobility is higher for B magnitudes between 2 and 6 T, whereas this influence decreases for B of 8 T and 10 T. To provide an explanation for this trend, it is proposed that increasing the magnetic dose initially influences the analyzed parameters. As B increases, an energy saturation point is reached where the mentioned effects decrease in the B range of study.

Furthermore, experimental and computational studies of aquaporins have reported single-channel osmotic permeabilities in the range of 0.5–16 × 10⁻^14^ cm^3^ s⁻^1^, depending on the specific isoform and simulation protocol (Zhu et al. 2004; Mamonov et al. 2007; Hashido et al. 2007). The pf values obtained in the present study fall within this range, confirming that the calculated magnitudes are biophysically reasonable.

### Water Flux Through AQP Exposed to Magnetic Fields with B of 100 kT

**Fig. 9:**
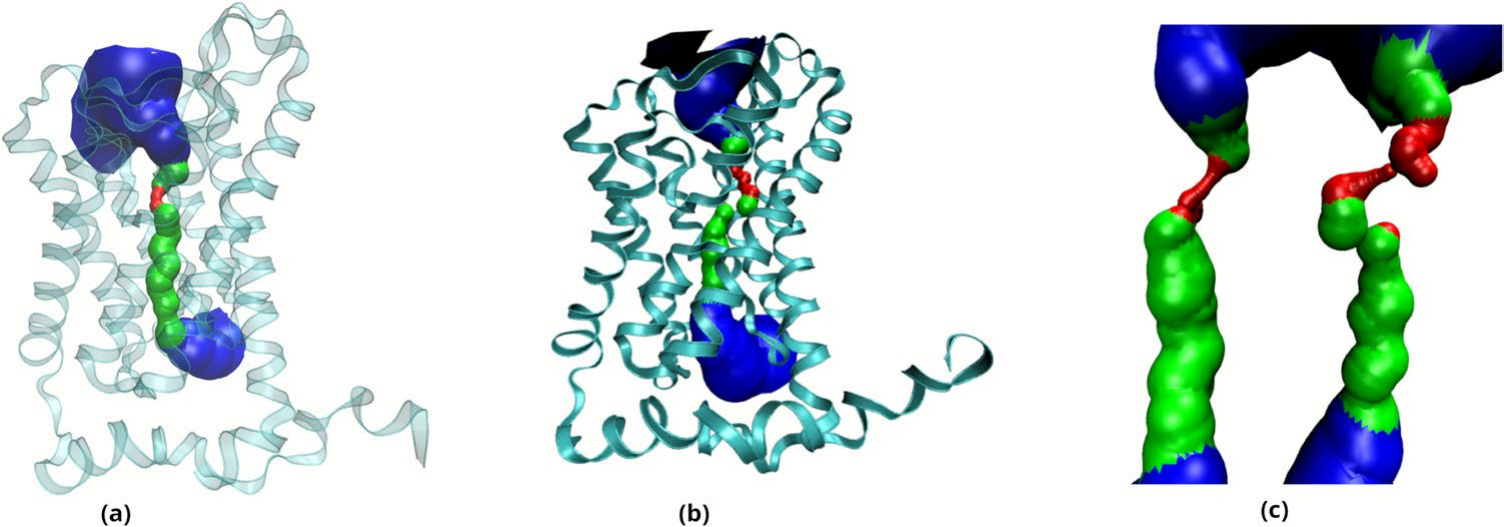
Mean pore profile inside the protein channels. **a** Without magnetic field exposure and **b** exposed to a field with B of 100 kT. In **c** both pores are shown side by side for easy comparison, on the left the pore of the system without magnetic field exposure and on the right the one exposed to B of 100 kT

Exposure to the 100 kT magnetic field induced pronounced changes in the conformational dynamics of TIP3;1. The average pore profile (see Fig. 9) demonstrates a clear distinction between the control system and the system exposed to 100 kT, indicating a more pronounced channel bottleneck under the 100 kT magnetic field.

**Fig. 10:**
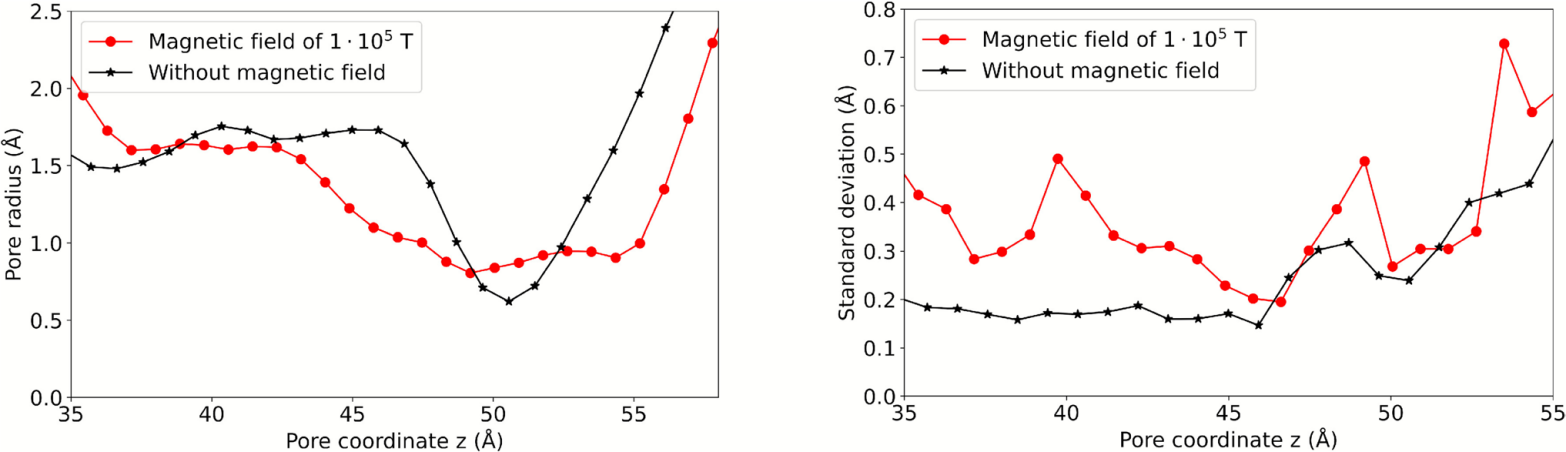
Average pore radius r (**a**) and standard deviation of r (**b**) of TIP3;1 for B of 0 T and 100 kT

The average pore radius, as depicted in Fig. 10a, quantitatively validates the augmentation in the length of the bottleneck and the single-file water channel. This phenomenon can be elucidated by the influx of a greater amount of energy, which escalates the conformational variability, modifies the pore surface area, and alters the water displacement pathway across the membrane. This increased conformational variability is confirmed by the increase in the standard deviation of the pore radius under B of 100 kT (Fig. 10b). Exposure to B of 100 kT also significantly affected water mobility, recording a p_f_ of (2.654 ± 0.12)·10^–14^ cm^3^ s^−1^, compared to (0.634 ± 0.052)·10^–14^ cm^3^ s^−1^ in the control system, indicating a considerable increase in water mobility across the membrane fragment.

### Magnetic Dose Considerations in Molecular Dynamics Studies

Despite the considerations discussed above, experimental conditions in magnetobiology and those in molecular dynamics (MD) simulations differ fundamentally. Typical experiments apply B ranging from 1 mT to 1 T over periods of minutes, hours or days, whereas MD simulations operate on nanosecond timescales (Torres et al. 2018; Vashisth and Nagarajan 2009; Balakrishnan et al. 2023; Sulong et al. 2022). As shown in Table 1, magnetic field intensities above 100 kT are required in simulations to match the magnetic dose delivered in experimental setups.

**Table 1:** Magnetic dose as a function of** B** and exposure time, according to Pietruszewski, 2015 (Pietruszewski and Martínez 2015)

B (T)	Exposure time (s)	Magnetic Dose (J *s*m⁻^3^)
0.150	3600	32,050,000
1	10^–8^	0.003958
100 000	10^–8^	39,580,000

The first row represents a typical experimental setup, with a **B** of 150 mT and an exposure time of one hour

Furthermore, even when magnetic doses are comparable, there is a considerable spatial disparity: biological systems, such as seeds, occupy volumes on the scale of cubic centimeters, while simulation boxes are constrained to nanometer-scale volumes. This spatial discrepancy precludes a direct analogy between the two approaches. Therefore, the results obtained in this study demonstrate only the existence of magnetic field effects on water transport through aquaporins, without establishing a direct correlation with experimental findings. The primary goal here is to reveal how magnetic fields influence water permeability and to elucidate the molecular mechanisms that might underlie magnetoreception. Consequently, we propose that magnetic fields can modulate cellular water flow and may act as one of the contributing factors influencing seed germination in magnetically treated seeds. However, this hypothesis cannot be definitively tested using molecular dynamics simulations alone.

Although this comparison is purely energetic and ignores macroscopic heterogeneities, it provides a rational basis for exploring stronger fields in silico to approximate the total magnetic dose delivered over experimental timescales. We explicitly stress that the 100 kT condition is exploratory and not intended to represent a biologically accessible field strength.

### Perspectives and Biological Implications

The impact of a magnetic field on water flow through TIP3;1 in systems exposed to an external magnetic field ranging from 0 to 10 T, and to a magnetic field with a strength of 100 kT, has been observed. The resulting changes manifest in the dynamics of protein conformations, the intermolecular interactions within the channel and the water mobility across the membrane fragment. It is noteworthy that these effects are interconnected; variations in pore size can limit or facilitate water flow by influencing the amount of hydrogen bonds in the region. In turn, these variations in intermolecular interactions have the potential to modulate the freedom of water movement.

Accordingly, one plausible mechanism of seed magnetoreception is the influence of the field on water flow through TIP3;1, which can lead to alterations in the other physiological and biological processes in which this AQP participates. Therefore, increased water mobility through the tonoplast has the potential to increase the hydrolysis of inactive forms of gibberellins stored in the vacuole, which triggers germination in a shorter time as observed experimentally. The findings suggest that magnetic fields can modulate water transport through aquaporins. Such modulation may, in turn, influence physiological processes that depend on rapid water movement, such as seed germination.

Within the broader framework of the Magnetobiology Research Group at the University of Caldas, these results provide computational support for the hypothesis that magnetically treated seeds exhibit increased GA₃ levels due to enhanced vacuolar water flow. Beyond aquaporins, magnetic fields may also affect auxiliary signaling pathways, for example, by modulating Ca^2^⁺ flux through the plasma membrane, which regulates Ca^2^⁺-dependent protein kinases responsible for aquaporin activation, or by influencing enzymatic activities such as amylases involved in seed metabolism. Further computational and experimental work will be needed to clarify these interactions.

## Conclusions

The present study explored the influence of external magnetic fields on water transport through *Zea mays* L. TIP3;1 aquaporins using molecular dynamics simulations with a modified version of GROMACS that includes Lorentz-type magnetic effects. The results demonstrate that magnetic fields in the range of 2–6 T enhance pore-radius variability and increase osmotic permeability (p_f_), indicating that moderate fields can promote conformational flexibility and facilitate water transport. At higher intensities (8–10 T), these effects attenuate, suggesting a saturation behavior in the response of the system.

The exploratory simulation performed at 100 kT further supports this interpretation by showing amplified conformational fluctuations and a notable increase in water mobility. This condition does not represent any realistic biological exposure but serves to approximate the magnetic dose corresponding to prolonged low-field treatments in experimental systems. The computed p_f_ values fall within the biophysical range reported for other aquaporins, confirming the plausibility of the observed magnitudes. These findings provide a molecular-level explanation consistent with experimental evidence showing enhanced seed germination and hormone activation under magnetic treatment.

Overall, the results suggest that magnetic fields can modulate aquaporin-mediated water transport by altering protein flexibility and hydrogen-bond dynamics. While these conclusions are qualitative and exploratory, they highlight a possible mechanistic link between magnetic fields and cellular water transport processes. Future computational and experimental studies should aim to refine this mechanism under realistic field strengths and exposure durations, contributing to a deeper understanding of plant magnetoreception and its potential agronomic applications.

## Supplementary Information

Below is the link to the electronic supplementary material.

Supplementary file1 (DOCX 14 kb)

## Data Availability

All data supporting the findings of this study are available within the paper and its Supplementary Information (SI).
