# Supplementary material for: Analysis of the Effect of the Magnetic Field on Water Flux Through TIP3;1 Aquaporins Using Molecular Dynamics in GROMACS
Source: J Membr Biol. 2026 Feb 5;259(1):7. doi: 10.1007/s00232-025-00368-x (PMC12876521; doi:10.1007/s00232-025-00368-x)
Supplement: Supplementary file 1 — Supplementary file1 (DOCX 14 kb) [file 232_2025_368_MOESM1_ESM.docx]

**Analysis of the effect of the magnetic field on water flux through TIP3;1 aquaporins using molecular dynamics in GROMACS**

*Diego Fernando Nieto-Giraldo^*[a]^ , José Mauricio Rodas Rodríguez^[a]^, Javier Torres-Osorio^[b]^*

a] Department of Chemistry, Universidad de Caldas. Calle 65 # 26-10, Manizales-Caldas, Colombia. * diego.nieto@ucaldas.edu.co

[b] Department of Physics, Universidad de Caldas. Calle 65 # 26-10, Manizales-Caldas, Colombia.

## ****S1. Extended Simulation Setup****

### ****S1.1. Force field and MD engine****

All simulations were performed with a **custom-modified version of GROMACS 2023.2**, which incorporates an additional magnetic force term within the **Velocity–Verlet (md-vv) integrator**, allowing computation of the Lorentz-type magnetic force:

F_i_ =q_i_(v_i_×B)

Only the integrator was modified; the CHARMM36 force field and SPC/E water model remain unchanged and contain **no magnetic-specific parameters**. Full implementation details are described in Nieto-Giraldo et al., RSC Advances (2025).

### ****S1.2. Magnetic field setup****

Because the magnetic term is not yet implemented as an .mdp parameter, the field intensity must be hard-coded. For each condition (0, 1, 2, 4, 6, 10 T, and 100 kT):

1. The value of **B** was manually set in propagator.cpp as described in the reference above.
2. GROMACS was **recompiled** after each modification.
3. Simulations were run using **integrator = md-vv**, the only integrator compatible with the modified magnetic scheme.

This procedure is consistent with the current developmental state of the magnetic implementation.

### ****S1.3. Initial minimization and equilibration****

Energy minimization used steepest descent until reaching a maximum force < 1000 kJ·mol⁻¹·nm⁻¹.
A simulated annealing stage (NVT + semi-isotropic NPT) heated the system from 0 to 303 K.

### ****S1.4. Production runs****

Production MD runs used the parameters listed in Section S2 (md.mdp).
Two independent replicates were run for each magnetic field intensity, differing only in initial Maxwell–Boltzmann velocities.

### ****S1.5. System size****

The final assembled system contained approximately 215,000 atoms, including protein, lipids (POPC/POPE/CHL1), SPC/E water, and counterions (Cl⁻). Box dimensions after equilibration were approximately **15 × 15 × 9 nm³**.

# ****S2. GROMACS Input Files****

The full .mdp files used for minimization, equilibration, and production simulations are provided as part of the Supplementary Dataset and archived in the public repository.

Below we reproduce the text of these files for reproducibility.

## ****S2.1. Minimization (minim.mdp)****

integrator = steep ; Algorithm (steep = steepest descent minimization)

emtol = 1000.0 ; Stop minimization when the maximum force < 1000.0 kJ/mol/nm

emstep = 0.01 ; Energy step size

nsteps = 50000 ; Maximum number of (minimization) steps to perform

nstlist = 1 ; Frequency to update the neighbor list and long range forces

ns_type = grid ; Method to determine neighbor list (simple, grid)

rlist = 1.2 ; Cut-off for making neighbor list (short range forces)

coulombtype = PME ; Treatment of long range electrostatic interactions

rcoulomb = 1.2 ; Short-range electrostatic cut-off

rvdw = 1.2 ; Short-range Van der Waals cut-off

pbc = xyz ; Periodic Boundary Conditions

## ****S2.2. Annealing / NVT (nvt.mdp)****

integrator = md-vv ;

dt = 0.002 ; 2 fs

nsteps = 2500000 ;

; Bond parameters

continuation = no ; starting up

constraints = all-bonds ; constrain all bond lengths

constraint-algorithm = lincs ; holonomic constraints

lincs-iter = 1 ; accuracy of LINCS

lincs-order = 4 ; also related to accuracy

; Output control

nstxout = 1000 ; save coordinates every 2 ps

nstvout = 1000 ; save velocities every 2 ps

nstfout = 1000 ; save forces every 2 ps

nstenergy = 1000 ; save energies every 2 ps

; Neighborsearching

nstlist = 5 ; 10 fs

ns_type = grid ; search neighboring grid cells

rlist = 1.2 ; short-range neighborlist cutoff (nm)

refcoord_scaling = all

rcoulomb = 1.2 ; short-range electrostatic cutoff (nm)

rvdw = 1.2 ; short-range van der Waals cutoff (nm)

; Electrostatics

coulombtype = PME ; Particle Mesh Ewald for long-range electrostatics

pme_order = 4 ; cubic interpolation

fourierspacing = 0.16 ; grid spacing for FFT

; Temperature coupling is on in three groups

Tcoupl = v-rescale ; Weak coupling

tc_grps = SISTEMA1 SISTEMA2 ; two groups - more accurate

tau_t = 0.1 0.1 ; time constant, in ps

ref_t = 303 303 ; reference temperature, one for each group, in K

; Pressure coupling

Pcoupl = Berendsen ; Weak coupling

Pcoupltype = semiisotropic ; uniform scaling of x-y vectors, independent z

ref_p = 1.0 1.0 ; reference pressure, x-y, z (in bar)

compressibility = 4.5e-5 4.5e-5 ; isothermal compressibility, bar^-1

; Periodic boundary conditions are on in all directions

pbc = xyz ; 3-D PBC

; Dispersion correction

DispCorr = EnerPres ; account for cut-off vdW scheme

; COM motion removal

; These options remove motion of the protein/bilayer relative to the solvent/ions

nstcomm = 1

comm-mode = Linear

comm-grps = SISTEMA1 SISTEMA2

; Simulated annealing

annealing = single single ; single sequence of points for each T-coupling group

annealing_npoints = 2 2 ; two points - start and end temperatures

annealing_time = 0 100 0 100 ; time frame of heating - heat over period of 500 ps

annealing_temp = 0 303.15 0 303.15 ; start and end temperatures

## ****S2.3. Production (md.mdp)****

; Run parameters

integrator = md-vv ; leap-frog integrator

nsteps = 2500000 ;

dt = 0.002 ; 2 fs

; Output control

nstxout = 1000 ; save coordinates every 2 ps

nstvout = 1000 ; save velocities every 2 ps

nstxtcout = 1000 ; xtc compressed trajectory output every 2 ps

nstenergy = 1000 ; save energies every 2 ps

nstlog = 1000 ; update log file every 2 ps

; Bond parameters

continuation = yes ; Restarting after NPT

constraint_algorithm = lincs ; holonomic constraints

constraints = all-bonds ; all bonds (even heavy atom-H bonds) constrained

lincs_iter = 1 ; accuracy of LINCS

lincs_order = 4 ; also related to accuracy

lincs_warnangle = 60

; Neighborsearching

ns_type = grid ; search neighboring grid cels

nstlist = 5 ; 10 fs

refcoord_scaling = all

rlist = 1.2 ; short-range neighborlist cutoff (in nm)

rcoulomb = 1.2 ; short-range electrostatic cutoff (in nm)

rvdw = 1.2 ; short-range van der Waals cutoff (in nm)

; Electrostatics

coulombtype = PME ; Particle Mesh Ewald for long-range electrostatics

pme_order = 4 ; cubic interpolation

fourierspacing = 0.13 ; grid spacing for FFT

; Temperature coupling is on

tcoupl = v-rescale ; More accurate thermostat

tc-grps = SISTEMA1 SISTEMA2 ; two coupling groups - more accurate

tau_t = 2 2 ; time constant, in ps

ref_t = 303.15 303.15 ; reference temperature, one for each group, in K

; Pressure coupling is on

pcoupl = Parrinello-Rahman ; Pressure coupling on in NPT

pcoupltype = semiisotropic ; uniform scaling of x-y box vectors, independent z

tau_p = 4 ; time constant, in ps

ref_p = 1.0 1.0 ; reference pressure, x-y, z (in bar)

compressibility = 4.5e-5 4.5e-5 ; isothermal compressibility, bar^-1

; Periodic boundary conditions

pbc = xyz ; 3-D PBC

; Dispersion correction

DispCorr = EnerPres ; account for cut-off vdW scheme

; Velocity generation

gen_vel = no ; Velocity generation is off

; COM motion removal

; These options remove motion of the protein/bilayer relative to the solvent/ions

nstcomm = 1

comm-mode = Linear

comm-grps = SISTEMA1 SISTEMA2

# ****S3. Preparation of Skipped Trajectories****

To reduce file sizes for public sharing, trajectories were downsampled using:

gmx trjconv -s run.tpr -f run.xtc -o run_skip10.xtc -skip 10

This stores **1 of every 10 frames**, reducing size by a factor of 10. No center-of-mass corrections, fitting, or PBC manipulations were applied. The repository includes skipped trajectories for all replicates and magnetic field treatments.

## ****S4. Analysis Scripts****

All scripts used to extract structural and dynamical quantities from the trajectories are provided in the public repository associated with this article. These scripts perform data extraction only (generation of .csv files) and do not carry out statistical analysis or plotting; therefore, the plotting routines are not included here.

### ****S4.1. Pore Radius Extraction (HOLE2 + MDAnalysis)****

The radius profile of each monomer was computed using a Python script that interfaces MDAnalysis with the HOLE2 executable.
The script:

1. loads the .pdb and .xtc trajectory;
2. identifies the monomer of interest;
3. runs HOLE2 frame by frame;
4. extracts the radius at each z-position;
5. calculates the mean radius and standard deviation across frames;
6. exports the results into a .csv file containing the columns:
   - **z-coordinate**,
   - **average pore radius**,
   - **mean standard deviation**.

The script used for this step (radious.py) is fully available in the repository.

### ****S4.2. Hydrogen-Bond Extraction (MDAnalysis)****

Hydrogen bonds between channel-lining residues and water molecules inside the single-file region were evaluated using MDAnalysis' HydrogenBondAnalysis.

The script:

1. selects donor/acceptor groups based on atom types;
2. computes hydrogen bonds for every frame;
3. bins the hydrogen-bond counts along the channel axis (z);
4. outputs a .csv file containing:
   - **z-coordinate**,
   - **number of hydrogen bonds**.

The code (Hbond.py) is also provided in the repository.

### ****S4.3. Post-processing of extracted data****

All .csv files generated by the scripts were subsequently analyzed and visualized using Python’s Matplotlib library.

# ****S5. Water Diffusion Coefficient and pf Calculation****

## ****S5.1. Extraction of diffusion coefficient (Dn) with VMD****

The diffusion coefficient along the channel axis was computed using the **Diffusion Coefficient Tool plugin** in **VMD 1.9.3.**

### ****Procedure:****

1. The GROMACS trajectory (.xtc) and structure (.pdb) were loaded in VMD.
2. Using tools such as atom selection syntax, water molecules in the single-file region were selected:
   - region delimited from HOLE2 profiles
   - optionally separated by subchannels (Figure 8 in main text)
3. The plugin was configured to:
   - compute diffusion along **the z-axis only** (consistent with single-file transport)
   - restrict analysis to molecules within the pore region
   - calculate MSD(τ) for each water molecule
4. The plugin automatically performs a linear fit of the MSD curve in the diffusive regime and outputs:
   - diffusion coefficient **Dn** (Å²/ps)

No additional scripting was required, as the plugin performs all calculations.

## ****S5.2. Conversion from D_n_ to p_f_****

Following Zhu (2004) and Hashido (2007), the osmotic permeability coefficient was computed as:

p_f_ =v_w_D_n_

where:

- D_n_ is the 1D diffusion coefficient obtained from VMD (converted to cm²/s),
- v_w_ is the molecular volume of water.

Because this formula is linear, no additional scripts were required. The computation was performed in a spreadsheet environment.

# ****S6. Data and Code Availability****

The following items have been deposited in the public repository (https://github.com/DiegoNieto0/Water_flux_AQP_magnetic_MD_GROMACS):

- All .mdp input files (minim, NVT/annealing, production)
- All analysis scripts (Hbond.py, radious.py)
- Skipped trajectories (*.xtc with stride 10) for each replicate
- Final structures (*.gro)

All MD input files, analysis scripts, and skipped trajectories are publicly available at:

https://github.com/DiegoNieto0/Water_flux_AQP_magnetic_MD_GROMACS
